# Cardiac toxicity after intraurethral instillation of lidocaine: A case report and review of literature

**DOI:** 10.1590/S1677-5538.IBJU.2019.0259

**Published:** 2020-01-10

**Authors:** Mohamad Moussa, Mohamed Abou Chakra

**Affiliations:** 1 Department of Urology Zahra University Hospital Beirut Lebanon Department of Urology, Zahra University Hospital, Beirut, Lebanon;; 2 Department of Urology Faculty of Medical Sciences Lebanese University Beirut Lebanon Department of Urology, Faculty of Medical Sciences, Lebanese University, Beirut, Lebanon

## INTRODUCTION

Local anesthetics are used in a wide range of clinical situations to prevent acute pain and to stop or ameliorate pain produced by cancer and chronic conditions. Local anesthetics may have similar chemical structures, but different pharmacokinetic properties (1). Outpatient flexible cystoscopy is a common procedure done in outpatient clinics and is usually associated with some discomfort. In current clinical practice, 2% lidocaine gel is widely used as a local anesthetic lubricant before various forms of transurethral instrumentation (urethral catheterization, flexible or rigid cystoscopy, urethral dilatation) (2). Systemic local anesthetic toxicity is infrequent, it can lead to neurological and cardiac complications. The estimate of clinically important local anesthetic toxicity is 7.5 to 20 occurrences per 10.000 peripheral nerve blocks and approximately four occurrences per 10.000 epidurals (3).

## CASE REPORT

A 65- year-old male known to have T1G3 transitional papillary carcinoma of the bladder diagnosed three months ago treated by transurethral resection of the tumor followed by intravesical Bacillus Calmette-Guérin (BCG) therapy of 6 doses, presented for flexible cystoscopy for control in the outpatient clinic of the hospital. He has a negative past medical history. He has no known food or drug allergies. On pre-procedure assessment, his blood pressure was 110/80mmHg, heart rate 72 beats per minute (bpm), respiratory rate 13 per minute and temperature 37.1ºC.

**Figure 1:**
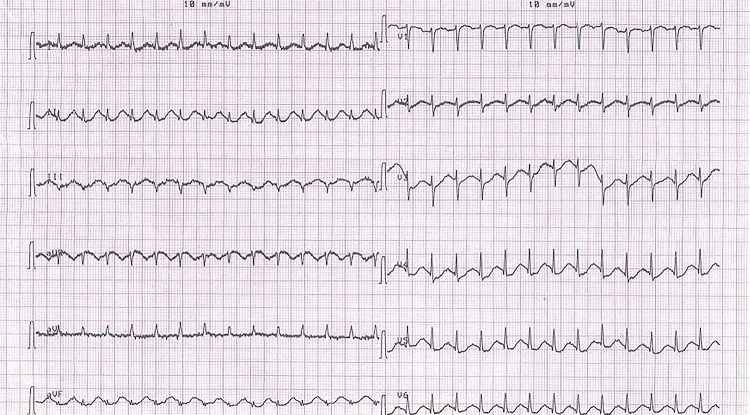
A 12-lead electrocardiogram (EKG) showing a regular narrow complex supraventricular tachycardia at 167 beats per minute (bpm) that occurred after intraurethral lidocaine injection.

In the supine position, prepping and draping in the usual sterile manner were done. Twenty mL of 2% lidocaine gel was applied intraurethrally followed by application of a penile clamp across the distal penis. After 5 minutes, the patient started to experience difficulty breathing and palpitations. Upon suspicion of local anesthetic toxicity, the rapid response team was informed promptly about the case. When arrived at the clinic, the patient was attached to the monitor and two IV lines were inserted. Adequate oxygenation by a face mask was initiated. Examination revealed a heart rate of 160bpm, blood pressure of 180/100mmhg and respiratory rate of 22 per minute. ECG showed supraventricular tachycardia with a rate of 167/min (Figure-1). The patient was given immediately a 100mL bolus of 20% intravenous lipid emulsion (intralipids) over 3 minutes followed by 200mL infusion over 15min. Restoration of normal sinus rhythm was observed within 5 minutes of injection. Vital signs returned within the normal limits: blood pressure 120/90mmhg, heart rate 78bpm, respiratory rate 16per minute. The patient was admitted to CCU for monitoring. Blood examinations showed a normal troponin T level, CPK, CPK-MB, metabolic panel. Two days later, the patient was discharged and his clinical status was stable on follow-up.

## DISCUSSION

Cardiovascular collapse from accidental local anesthetic toxicity is infrequent. Cardiac toxicity of local anesthetics follows a biphasic pathway, at lower concentrations, sympathetic nervous system activation during the CNS excitatory phase can lead to hypertension and tachycardia. Then, a myocardial depression, conduction block and decreased autonomic flow occur (3, 4).

In a review of systemic toxicity cases from 1979 to 2009, Di Gregorio et al. studied the clinical presentation of local anesthetic systemic toxicity. They concluded that 60% of the patients follow the classic pattern that includes progressive worsening neurologic symptoms occurring shortly after the injection of local anesthetic and paralleling progressive increases in blood local anesthetic concentration then seizures and coma and in extreme cases cardiovascular collapse. In other cases, delayed symptoms can develop or only signs of cardiovascular toxicity without nervous system toxicity. Cardiovascular toxicity characteristics were bradycardia/asystole, tachycardia, hypotension, wide complex, ST-segment changes, ventricular tachycardia and ventricular fibrillation (5).

The risk factors for local anesthetic systemic toxicity are: extremes of age, hepatic dysfunction, low cardiac output states, cardiac pathology, pregnancy, use of β-blocker, digoxin, calcium antagonists (6).

The pathophysiology of local anesthesia systemic toxicity is difficult to discern with the lack randomized trials assessing this point. Most theories are based upon which binding site, ion channel, signaling pathway, are involved in CNS and cardiac toxicity (7). The principal mechanism of cardiac toxicity relates to the blockade of myocardial voltage-dependent sodium channels, which leads to an increase in the PR interval and QRS duration, while persistent sodium channel blockade predisposes to re-entrant arrhythmias (3). De La Coussaye demonstrated that bupivacaine, more potently than ropivacaine alters ventricular conduction via the His-bundle (8).

The diagnosis of lidocaine toxicity is usually clinical as serum levels are not readily available, old studies suggests plasma concentrations above 5μgmL^1^ were associated with neurological symptoms and levels above 10μgmL^1^ with cardiovascular instability (9).

Chang et al. reported a case of cardiac arrest immediately following intraurethral administration of lidocaine for cystourethroscopy in an 87-year-old man with intraurethral mucosa lesions from previous attempts of Foley catheter insertion (10). Clapp et al. reported a case of a 2.5-year-old-girl who suffered of a generalized tonic-clonic seizure secondary to the intravesical instillation of lidocaine for the symptomatic relief of postoperative bladder spasm (11).

Initial management of local anesthesia cardiac toxicity should be focused on airway management and circulatory support. The management is symptomatic to prevent hypoxia, acidosis, and hyperkalemia, which may increase the risk of cardiac toxicity (12). Management of local anesthetic-induced cardiac arrest is focused on restoring cardiac output with standard ACLS guidelines with a few adjustments such as small doses of epinephrine (less than 1 microgram/kg) (13).

Intravenous infusion of a lipid emulsion has become part of the treatment for systemic toxicity from local anesthesia, it can be used to treat cardiovascular and central nervous system failure (14). American Society of Regional Anesthesia and Pain Medicine recommends for patients who weigh less than 70kg an initial bolus of 1.5mg/kg of 20% intravenous lipids over 2-3 minutes followed by a continuous infusion of 0.25mL/kg/minute. If hemodynamic stability is not achieved, repeating the bolus dose (up to a total of 3 boluses, 3 to 5 min. apart), followed by increasing the infusion (to 0.5mL/kg/min) is recommended. Infusion should be continued for at least 10 min. after circulatory stability is attained (15).

The exact mechanism of action of lipid emulsions in the treatment of local anesthesia systemic toxicity is not completely known. Several theories are proposed, among which the ‘‘lipid sink theory’’. This theory postulates that the administration of a lipid emulsion provides an alternative binding surface and therefore acts as a ‘‘sink’’ for the fraction of local anesthesia molecules rendering them ineffective (16). Other studies suggested that lipid emulsions can contribute to the enhanced redistribution and delivery of local anesthetics into lesser occupied or perfused sites of deposition/metabolism (adipose tissue, muscle, liver) which has been described as a lipid shuttle theory. In addition to the ‘‘lipid sink and shuttle’’ theories, other mechanisms involved in lipid emulsion resuscitation are mainly: fatty acid supply, a reversal of mitochondrial dysfunction, inotropic effect, GSK-3β (Glycogen synthase kinase-3β) phosphorylation inhibition of nitric oxide release and reversal of cardiac sodium channel blockade (17).

In our case, the patient had no history of heart disease and was not taking any medications that increase the risk of local anesthesia toxicity. He has no evident urethral injury that could increase the absorption of lidocaine beside transurethral resection of bladder tumor and BCG (Bacillus Calmette-Guérin) instillation of 6 times.The lesson learned is the need for the availability of lipid emulsion therapy in every emergency settings whenever local anesthesia is used. This therapy led to successful management of the arrhythmia. The cardiac toxicity after intraurethral administration of lidocaine is documented in only one case in the literature (10). It can occur as isolated toxicity as described in a recent review of published cases where one-fifth of LAST episodes presented with isolated cardiovascular system disturbances (18). Further investigations are needed to clarify the risks and etiologies.

## CONCLUSION

Systemic toxic effects after local lidocaine application are infrequent and could be life-threatening. The rapid identification of clinical symptoms is key to prevent mortality. The majority of data in the literature suggest that intravenous lipid emulsion is effective for reversing cardiovascular toxicity. Further basic and clinical studies seem to be indispensable to establish more effective treatment guidelines of cardiac arrhythmia induced by local anesthesia.
